# Special Issue: New Insights into Protein Glycosylation

**DOI:** 10.3390/molecules28073263

**Published:** 2023-04-06

**Authors:** Yuuki Kurebayashi, Hideyuki Takeuchi

**Affiliations:** Department of Biochemistry, School of Pharmaceutical Sciences, University of Shizuoka, Shizuoka 422-8526, Japan; kurebayashi@u-shizuoka-ken.ac.jp

Protein glycosylation is a general post-translational modification pathway that controls various biological functions including protein trafficking, cell adhesion, and protein-ligand interaction. Most of the proteins expressed on the cell surface or secreted extracellularly are glycoproteins. Glycans on proteins show highly complex patterns. However, on the other hand, glycan modifications are under precise control, and disorders of glycosylation on proteins cause various diseases [1,2,3]. Following polypeptide folding, protein glycosylation such as *N*-glycosylation and *O*-glycosylation occurs in the endoplasmic reticulum and Golgi apparatus. Glycans on proteins work as ligands for glycan-binding molecules such as lectin [4,5], as well as for quality control and the functional regulation of proteins [6]. Specific interactions between glycans and glycan-binding molecules play critical roles in intracellular and extracellular biological processes, and even host–pathogen interaction. Slight differences in the glycan structure can have a significant impact on these functions.

Glycan heterogeneity is an important issue in understanding the physiological roles of glycoproteins or protein glycosylation. Many glycoproteins show different glycan structures depending on the animal species or cell from which they are derived. In addition to that, even at a particular glycan attachment site on a protein synthesized by a particular cell type, a wide range of variations in glycan structures are most often observed. Glycans on proteins show a linear or branched chain-like structure that consists of monosaccharides linked by a covalent bond. The glycan structures on proteins are biosynthesized by the successive addition of monosaccharides by a glycosyltransferase in most cases. There are many types of glycosyltransferases that play an essential role in protein glycosylation, and each enzyme is responsible for the formation of specific glycan structures. In recent years, with the development of structural analysis technology for glycans, complex glycan structures on glycoproteins have been revealed one after another, and research on how the formation of these glycan structures is regulated has been widely conducted [7,8].

In this Special Issue, many novel findings in glycobiology, such as the regulation of protein functions by glycans and the properties of glycosyltransferases that control the biosynthesis of glycans, were reported. Several groups reported the study on atypical *O*-glycans that differ from typical *N*-glycans and *O*-glycans. Pennarubia et al. reported the results of a comparison of the glycan modulation function of three fringe (FNG) *N*-acetylglucosamine (GlcNAc)-transferases, responsible for the elongation of the *O*-fucose glycan on epidermal growth factor (EGF)-like repeats [9]. Three FNGs showed different activities towards *O*-fucose glycan on NOTCH1 in vitro. Mass-spectrometry-based glycoproteomics analysis revealed that Lunatic FNG (LFNG) has a higher enzymatic activity than Manic FNG (MFNG) and Radical FNG (RFNG). In addition, they showed that LFNG has a dominant effect over MFNG or RFNG on NOTCH1 activation in a cell-based Notch signaling assay. Barua et al. reported the potential roles for EGF domain-specific *O*-linked GlcNAc-transferase (EOGT) and LFNG in pancreatic cancers [10]. EOGT and LFNG are involved in the *O*-glycan modification on EGF-like repeats and the regulation of the ligand-dependent Notch signaling pathway. Bioinformatic analysis implied that the expression of EOGT and LFNG positively correlated with a subset of Notch signaling genes in pancreatic ductal adenocarcinoma. They demonstrated the loss of EOGT or LFNG, which impairs the cell proliferation and migration of Panc-1 cells.

In summary, this Special Issue showcases the recent impressive findings on the mechanisms that regulate the biosynthesis of various glycan structures and the biological functions of protein glycosylation. The structures of glycans and their roles are diverse, and their systematic understanding has not yet been achieved. A continuous and careful examination of individual glycans on proteins, regardless of their abundance, is expected to lead to the elucidation and regulation of the biological functions of protein glycosylation.
